# Early Life Nutrition and the Development of Offspring Metabolic Health

**DOI:** 10.3390/ijms23158096

**Published:** 2022-07-22

**Authors:** Deanne H. Hryciw

**Affiliations:** 1 School of Environment and Science, Griffith University, Nathan, QLD 4111, Australia; d.skelly@griffith.edu.au; 2 Griffith Institute of Drug Discovery, Griffith University, Nathan, QLD 4111, Australia; 3 Centre for Planetary Health and Food Security, Griffith University, Nathan, QLD 4111, Australia; 4 Institute for Health and Sport, Victoria University, Melbourne, VIC 3030, Australia

The developmental origins of health and disease (DOHaD) hypothesis describes the effects of parental perturbations around the periconception, pregnancy, and perinatal window that may lead to changes in offspring development and an increased risk of disease [1]. DOHaD, or simply “developmental programming”, refers to alterations in the physiology, metabolism, and epigenome of an offspring due to poor influences from the offspring’s father or mother [2]. It is well-established that the maternal in utero and early life (perinatal) environments are key to normal organ development and, thus, the disease risk in her offspring [3]. Importantly, more recent research has also determined that a poor paternal perinatal diet has the capacity to affect offspring development and disease risk [4]. The intention of this Special Issue is to explore the diverse recent research, which has showcased the importance of perturbations in maternal nutrition during pregnancy and early life, and the impact of this on the metabolic health of her offspring. Critical to this discussion are sex-specific differences in development and disease risk and the effects of a poor maternal environment on offspring health across the lifespan. Novel studies included in this Special Issue characterised the changes in metabolic health associated with maternal obesity, an elevated omega 6 diet, and elevated fructose diet and cannabis use. Finally, the role of the placental O-GlcNAc transferase in programming insulin sensitivity in offspring was investigated.

Excess dietary fructose is a major public health concern; in this context, the study by Smith et al. [5] investigated the developmental consequences of maternal fructose overconsumption on offspring health in guinea pigs. Excess dietary fructose consumed during pregnancy led to an increase in male and female triglycerides, palmitoleic acid, and total omega 7, with changes in mitochondrial metabolic activities of β-oxidation, electron transport chain and oxidative phosphorylation, and reactive oxygen species production. Smith et al. [5] revealed the high fructose maternal diet, programmed hepatic mitochondrial metabolism, and de novo lipogenesis in juvenile offspring, which is likely to predispose metabolic dysfunction later in life.

Gestational cannabis exposure has increased in recent times, despite limited evidence for its safety in the developing foetus. In the study by Oke et al. [6], prenatal exposure to Δ9-tetrahydrocannabinol, a component of cannabis, decreased the liver to body weight ratio at birth for her offspring, followed by catch-up growth by three weeks of age. In adult male offspring, there was increased visceral adiposity and higher hepatic triglycerides, suggesting that exposure to Δ9-tetrahydrocannabinol results in long-term dyslipidaemia associated with enhanced hepatic lipogenesis, driven by mitochondrial dysfunction and epigenetic modulation [6]. 

In the two manuscripts from Shrestha et al., the effects of a maternal high omega 6 (linoleic acid) diet during pregnancy and in the weaning/postnatal period were investigated in the adolescent [7] and adult [8] offspring. In adolescence, analysis of the metabolome identified changes in both sexes that were independent of the diet. In the female offspring, lysine concentrations were higher, while 3-hydroxybutyric acid and acetic acid were significantly higher in the male offspring [7]. In the adult offspring exposed to an elevated maternal and postnatal linoleic acid diet, postnatal linoleic acid decreased circulating omega 3 fatty acids and increased circulating omega 6 fatty acids in both sexes. However, maternal linoleic acid increased circulating leptin in females and decreased circulating adiponectin in males. Postnatal linoleic acid diet altered liver function, with sex-specific differences observed [8].

It is well-understood that maternal obesity increases the risk of health complications in offspring. In the study of Tajaddini et al. [9] in this Special Issue, the compounding effects of a poor offspring diet were investigated in rats. In the offspring, maternal obesity exacerbated the obesogenic phenotype produced by the postweaning cafeteria diet in male but not female offspring, with adiposity and liver gene expression compromised in males. These findings support the sex-specific detrimental effects of maternal obesity on offspring health outcomes, as well as the negative impacts of the postweaning diet. 

The placenta is key to organ development, and placental dysfunction can lead to foetal growth restriction, which is associated with perinatal morbidity and mortality. In the study by Moore et al. [10], placental O-GlcNAc transferase (OGT) was investigated due to its role as a marker and a mediator of placental insufficiency. In mice with a partial reduction in placenta-specific OGT, the effects of a high-fat maternal diet provided a metabolic challenge that revealed a decrease in body weight gain and an improved insulin tolerance for offspring, which was not observed when OGT was fully knocked out. Changes in body weight were not associated with changes in energy homeostasis in offspring. These changes may be due to an increased hepatic Akt phosphorylation in response to insulin treatment. Thus, it appears that placental OGT plays a role in peripheral insulin sensitivity [10].

This Special Issue also included three review articles that summarised the recent findings associated with maternal cannabinoid exposure [11], maternal melatonin and offspring cardiovascular development [12], and the gut microbiome and how this impacts the development of kidney disease [13].

In the review by Lee and Hardy [11], the impact of the endocannabinoid system on foetal development was discussed. These authors focused on the preclinical and clinical findings of the direct effects of exposure to cannabis and its constituents on fetal development and placental insufficiency, with some insight on the effects of activation of the endocannabinoid system on postnatal metabolic diseases.

In the review by Gombert and Codoñer-Franch [12], the role of melatonin, which is present in breast milk, on offspring circadian rhythm, inflammation, and gut microbiota was discussed. Gombert and Codoñer-Franch described the mechanisms by which melatonin from breast milk influences weight gain in infants, limiting the development of obesity and comorbidities in the long term. Collectively, these can modulate the ideal cellular environment for the development of the infant’s cardiovascular system [12].

Diabetic kidney disease (DKD) is a progressive disorder, which is increasing globally in prevalence due to the increased incidence of obesity and diabetes mellitus. Zaky et al. [13] discussed the role of the microbiome and reduced amounts of short-chain fatty acids in DKD development and progression. In their review, Zaky et al. [13] summarised the complex relationship between the gut microbiota and the kidney, and the role of gut dysbiosis in diabetes and obesity-related kidney disease [13].

The contributions of this Special Issue will provide some insight into the mechanisms which link a poor maternal environment and offspring metabolic health.
